# Mapping Pharmacological Network of Multi-Targeting Litchi Ingredients in Cancer Therapeutics

**DOI:** 10.3389/fphar.2020.00451

**Published:** 2020-04-24

**Authors:** Sisi Cao, Yaoyao Han, Qiaofeng Li, Yanjiang Chen, Dan Zhu, Zhiheng Su, Hongwei Guo

**Affiliations:** ^1^College of Pharmacy, Guangxi Medical University, Nanning, China; ^2^Key Laboratory of Longevity and Aging-related Diseases of Chinese Ministry of Education & Center for Translational Medicine, Guangxi Medical University, Nanning, China; ^3^School of Preclinical Medicine, Guangxi Medical University, Nanning, China; ^4^Department of Surgery, University of Melbourne, Parkville, VIC, Australia

**Keywords:** litchi, cancer, multi-ingredients, multi-targets, network pharmacology

## Abstract

Considerable pharmacological studies have demonstrated that the extracts and ingredients from different parts (seeds, peels, pulps, and flowers) of Litchi exhibited anticancer effects by affecting the proliferation, apoptosis, autophagy, metastasis, chemotherapy and radiotherapy sensitivity, stemness, metabolism, angiogenesis, and immunity *via* multiple targeting. However, there is no systematical analysis on the interaction network of “multiple ingredients-multiple targets-multiple pathways” anticancer effects of Litchi. In this study, we summarized the confirmed anticancer ingredients and molecular targets of Litchi based on published articles and applied network pharmacology approach to explore the complex mechanisms underlying these effects from a perspective of system biology. The top ingredients, top targets, and top pathways of each anticancer function were identified using network pharmacology approach. Further intersecting analyses showed that Epigallocatechin gallate (EGCG), Gallic acid, Kaempferol, Luteolin, and Betulinic acid were the top ingredients which might be the key ingredients exerting anticancer function of Litchi, while BAX, BCL2, CASP3, and AKT1 were the top targets which might be the main targets underling the anticancer mechanisms of these top ingredients. These results provided references for further understanding and exploration of Litchi as therapeutics in cancer as well as the application of “Component Formula” based on Litchi’s effective ingredients.

## Introduction

Cancer is one of the most serious public health problems globally. In 2018, approximately 18.1 million new cancer cases and 9.6 million cancer-related deaths occurred in the world (Bray et al., 2018). There is an urgent need for a more effective therapy. Traditional Chinese medicine (TCM) has been used for thousands of years in Asia for its good efficacy and compliance, and this also made it an important supplemental medicine in cancer treatment (Xiang et al., 2019). Comparing with the current “one drug, one target” mode, TCM has the feature of “multiple active ingredients, multiple targets” (Li and Zhang, 2013). Given that cancer is a complex disease which alters a range of cellular and molecular processes, TCM may hold the advantage of targeting multiple cancer-related molecules simultaneously with potential synergistic effects. However, as a result of the feature of “multiple ingredients, multiple targets”, herbs can potentially interact with prescription medications like when cancer patients use plant-based regimens with chemotherapy (Yeung et al., 2018; Parvez and Rishi, 2019; Pezzani et al., 2019). Therefore, the potential risk of using TCM as complementary medicine should be considered for maximum safety and efficacy.

*Litchi chinensis Sonn* (Litchi), a member of Litchi, Sapindaceae family, is a subtropical evergreen plant which has been widely cultivated as an economic cultivar for its delicious taste and rich nutrition fruitage in China, Philippines, Indonesia, and Vietnam (Mitra, 2002; Menzel et al., 2005). In China, Litchi seeds were used as an analgesic agent for the alleviation of neuralgia, orchitis, testicular swelling, hernia, gastralgia, lumbago, abdominal pain, etc. (Lan and Lan, 2011). The decoctions of Chinese herbal formula containing Litchi seeds were used as indigenous remedies for urologic neoplasms including prostate cancer, bladder cancer, and renal carcinoma (Shi, 2004; Wang, 2011c). Moreover, a considerable amount of studies have shown that in addition to Litchi seeds, the extracts and ingredients from other parts (peels, pulps, and flowers) of Litchi can exert multiple pharmacological actions which have the anti-inflammatory (Das et al., 2016), anti-oxidative (Lee et al., 2016), anti-bacterial (Yang et al., 2016), anti-viral (Gangehei et al., 2010; Xu et al., 2010a), anti-liver injury, and immune-enhancing effects (Noh et al., 2011; Huang et al., 2014a; Yamanishi et al., 2014; Huang et al., 2014b; Huang et al., 2016a; Su et al., 2016; Xiao et al., 2017; Queiroz et al., 2018). Furthermore, there was accumulating evidence indicating that the extracts and compounds from Litchi exhibit anticancer effects by targeting multiple proteins and signal pathways involved in cancer cell proliferation, metastasis, angiogenesis, apoptosis, autophagy, etc. However, current studies are limited to the traditional research method of identifying “single-drug, single-target, and single-pathway”, which failed to reflect the “multiple ingredients-multiple targets-multiple pathways” anticancer effects of Litchi. In order to elucidate its multiple modes of action, network pharmacology and bioinformatics were employed in this study as a powerful approach (Zhang et al., 2019a) to systematically analyze the complicated interactions between Litchi ingredients and confirmed targets based on published research results. This study has provided a solid base for the further exploration of its anticancer effects.

## Methods

**Figure 1:**
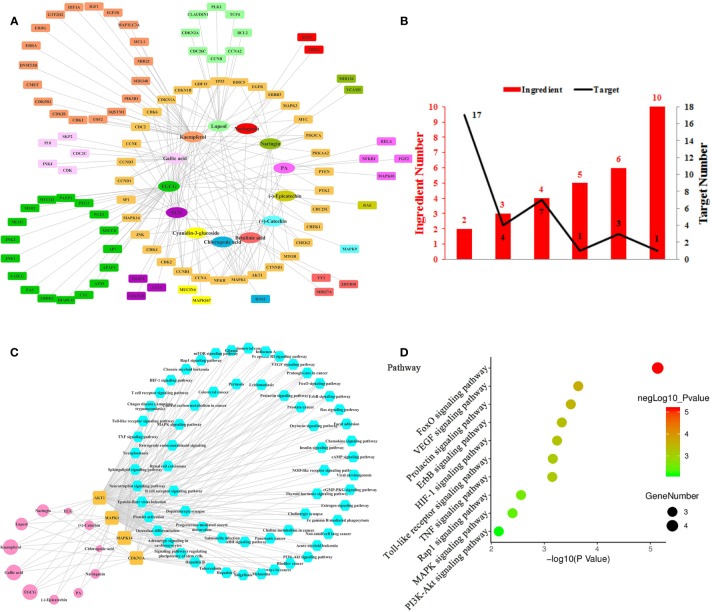
Ingredient-Target Network of Litchi Anti-Proliferation. **(A)** Network of 13 ingredients (oval) and 100 corresponding targets (rectangle). **(B)** The histogram of “ingredient and corresponding target number”. **(C)** The ingredient-top target-signal pathway network. The round rectangle nodes (orange) correspond to 4 top targets, ellipse nodes (pink) are 11 ingredients acting on top targets, the size of the nodes is illustrated from small to big in ascending order of degree values, the hexagon nodes (blue) represent signal pathways enriched based on top targets. **(D)** The top 10 pathway based on KEGG enrichment analysis.

**Figure 2:**
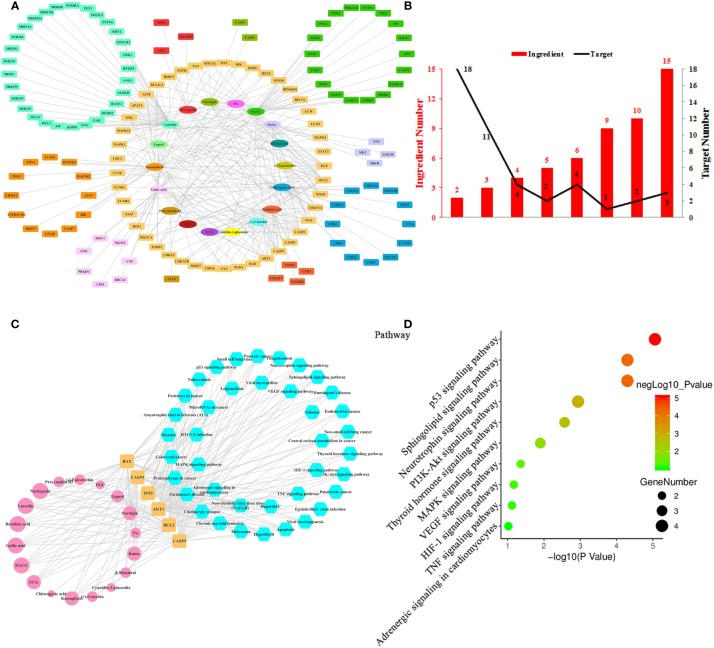
Ingredient-Target Network of Litchi Inducing Cancer Cell Apoptosis and Autophagy. **(A)** The ingredient-target network of 18 ingredients (oval) associated with 138 targets (rectangle). **(B)** The histogram of “ingredient and corresponding target number”. **(C)** The ingredient-top target-signal pathway network. The round rectangle nodes (orange) represent 6 top targets, ellipse nodes (pink) represent 18 ingredients acting on top targets, the size of the nodes is illustrated from small to big in ascending order of degree values, the hexagon nodes (blue) represent signal pathways enriched based on top targets. **(D)** The top 10 pathways based on KEGG enrichment analysis.

**Figure 3:**
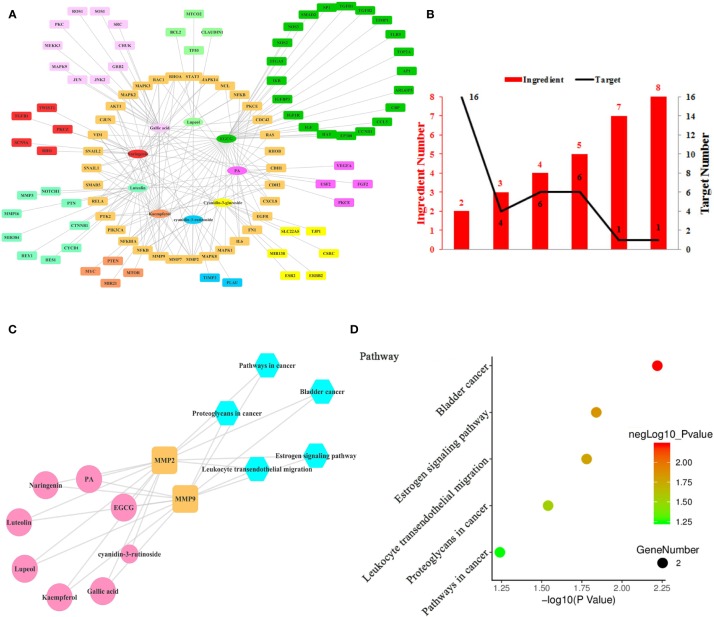
Ingredient-Target Network of Litchi Inhibiting Cancer Metastasis. **(A)** The ingredient-target network of 9 ingredients (oval) associated with 99 targets (rectangle). **(B)** The histogram of “ingredient and corresponding target number”. **(C)** The ingredient-top target-signal pathway network. The round rectangle nodes (orange) represent 2 top targets, ellipse nodes (pink) represent 8 ingredients acting on top targets, the size of the nodes is positively related to their degrees in the network, the hexagon nodes (blue) represent signal pathways enriched based on top targets. **(D)** The top 5 pathways based on KEGG enrichment analysis.

**Figure 4:**
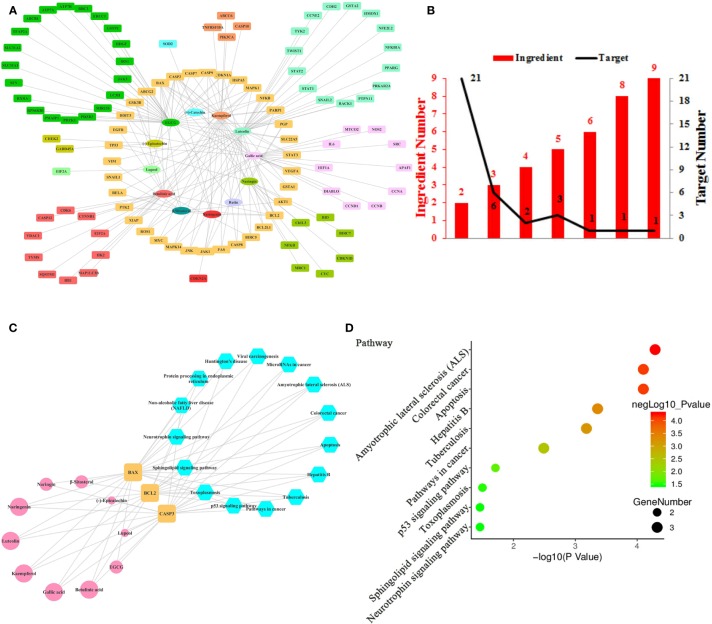
Ingredient-Target Network of Litchi Sensitizing Chemotherapy and Radiotherapy. **(A)** The ingredient-target network of 12 ingredients (oval) and 106 targets (rectangle). **(B)** The histogram of “ingredient and corresponding target number”. **(C)** The ingredient-top target-signal pathway network. The round rectangle nodes (orange) are top targets, ellipse nodes (pink) represent ingredients acting on top targets, the node size of ingredients is proportional to the degree values, the hexagon nodes (blue) represent signal pathways enriched based on top targets. **(D)** The top 10 pathways based on KEGG enrichment analysis.

**Figure 5:**
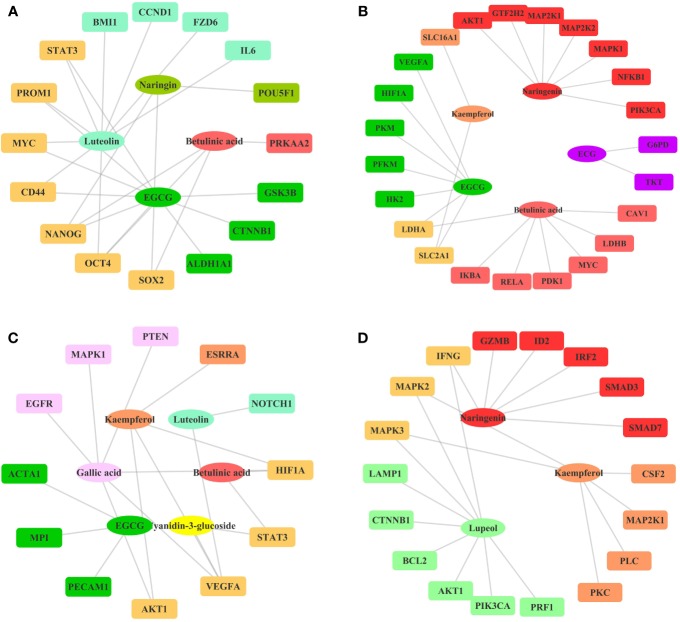
Ingredient-Target Network of Litchi Inhibiting Cancer Stemness, Metabolism, Angiogenesis, and Enhancing Immunity. **(A)** The ingredient-target network involving in cancer stemness. Different colors of ovals indicate 4 ingredients, the rectangle nodes represent 16 targets. **(B)** The ingredient-target networks involving cancer metabolism. Different colors of ovals indicate 5 ingredients, the rectangle nodes represent 23 targets. **(C)** The ingredient-target networks involving angiogenesis. Different colors of ovals indicate 6 ingredients, the rectangle nodes represent 12 targets. **(D)** The ingredient-target networks involving cancer immunity. Different colors of ovals indicate 3 ingredients, the rectangle nodes represent 18 targets.

We collected the anticancer ingredients and targets of Litchi based on original published articles. In order to systematically analyze the complex relationships between these anticancer ingredients and their targets, an interaction network was constructed by network pharmacology approach. All networks maps were visualized and analyzed by Cytoscape 3.2.1 (http://www.cytoscape.org/). As shown in the ingredient-target network (**Figures 1A**, **2A**, **3A**, **4A**, and **5**), the oval nodes represent ingredients, the rectangle nodes represent targets and each edge linking an ingredient to a target indicates a regulator-target relationship. In **Figures 1A**–**4A**, the targets distributing in the inner orange circle (rectangle) can be modulated by multiple ingredients rather than a single ingredient. The “degree” is an important parameter for the network pharmacology approach, which represents the number of related nodes to a particular node in the network. The greater the degree of a node, the more biologically important it is. Therefore, the top ingredients and targets were screened out by the Network Analyzer in Cytoscape based on the major parameter of “degree”. To further explore the core biological processes of the top targets involved, we performed KEGG pathway enrichment analysis (http://www.kegg.jp/) and screened out the top signal pathways based on the P-value. The relationships among top targets, corresponding ingredients and signal pathways were analyzed by combining Cytoscape 3.2.1 with KEGG pathway enrichment analysis. In order to test the reliability of the top ingredient-target interactions and explore the accurate binding modes, we performed molecular docking analysis by using surflex module of Sybyl X2.0. A total score greater than 6 represents good protein-ligand binding. The crystal structures of proteins (targets) were extracted from Protein Data Bank (https://www.rcsb.org/).

## Results

### Ingredients From Litchi

Litchi contains a variety of natural products, such as anthocyanins, flavonoids, phenolic acids, terpenes, fatty acids, sterols, lignans, coumarins, and esters. A total of 110 compounds (32 Anthocyanins, 32 Flavonoids, 9 Phenolic acids, 9 Tocotrienols, 8 Lignans, 4 Alcohols, 4 Sterols, 3 Triterpenes, 3 Fatty Acids, 2 Esters, 2 Glycosides, 1 Furfurals, 1 Coumarins) isolated from Litchi have been reported, which were summarized in **Table 1** according to the parts (peels, pulps, seeds, leaves, and flowers) of Litchi, with their molecular formulae, structure category and corresponding reference (Ref). As shown in **Table 1**, various kinds of chemical constituents were isolated from its peels (28 compounds), pulps (12 compounds), seeds (49 compounds) leaves (28 compounds), and flowers (1 compound). Among them, we identified flavonoids and anthocyanins which were mostly found in Litchi peels, seeds, and leaves to be the main compounds.

**Table 1:** Compounds Isolated from *L. chinensis*.

Parts	No	Ingredients	Formula	Compound yield (mg/100g)	Category	Ref
**Peels**	1	Cyanidin-3-rutinoside	C_27_H_31_O_15_	1.29–19.11	Anthocyanins	(Li et al., 2012)
	2	Cyanidin-3-glucoside	C_21_H_21_O_11_	0.80–1.80	Anthocyanins	(Li et al., 2012)
	3	Quercetin-3-glucoside	C_21_H_20_O_12_	5.00	Anthocyanins	(Ma et al., 2014)
	4	Malvidin-3-glucoside	C_23_H_25_O_12_	0.67–1.98	Anthocyanins	(Li et al., 2012)
	5	Epigallocatechin gallate (EGCG)	C_22_H_18_O_11_	/	Anthocyanins	(Xie, 2017)
	6	Dehydrodiepicatechin A	C_30_H_24_O_12_	0. 50	Anthocyanins	(Ma et al., 2014)
	7	Procyanidin A2	C_30_H_24_O_12_	68.30	Anthocyanins	(Sarni-Manchado et al., 2000)
	8	Proanthocyanidin A1	C_31_H_24_O_12_	0. 64	Anthocyanins	(Ma et al., 2014)
	9	Epicatechin-(4β→8,2β→O→7)-epicatechin-(4β→8)-epicatechin	C_45_H_36_O_18_	/	Anthocyanins	(Liu et al., 2007)
	10	Proanthocyanidin B2	C_30_H_26_O_12_	1.02	Anthocyanins	(Zhang et al., 2000)
	11	Proanthocyanidin B4	C_30_H_26_O_12_	0.48	Anthocyanins	(Zhang et al., 2000)
	12	Bis(8-epicatechinyl) methane	C_31_H_28_O_12_	0. 30	Anthocyanins	(Ma et al., 2014)
	13	8-(2-pyrrolidinone-5-yl)-(−)-epicatechin	C_19_H_14_O_7_N	0. 16	Anthocyanins	(Ma et al., 2014)
	14	(−)-epicatechin 8-C-β-D-glucopyranoside	C_21_H_20_O_11_	0. 08	Anthocyanins	(Ma et al., 2014)
	15	Naringenin7-O-(2,6-O-α-L-rhamnopyranosyl)-β-Dglucopyranoside	C_30_H_40_O_11_	0. 30	Anthocyanins	(Ma et al., 2014)
	16	(-)-Epigallocatechin (EGC)	C_15_H_14_O_6_	97.30	Anthocyanins	(Zhang et al., 2000)
	17	Rutin	C_27_H_30_O_16_	0. 44	Flavonoids	(Ma et al., 2014)
	18	Epiafzelechin	C_15_H_14_O_5_	/	Flavonoids	(Zhou et al., 2011)
	19	(-)-Epicatechin (EC)	C_15_H_14_O_6_	0. 22	Flavonoids	(Ma et al., 2014)
	20	(-)-Gallocatechin (GC)	C_15_H_14_O_7_	22.90	Flavonoids	(Zhang et al., 2000)
	21	Epicatechin glucoside	C_21_H_24_O_11_	/	Flavonoids	(Zhou et al., 2011)
	22	Kaempferol	C_15_H_10_O_6_	0. 33	Flavonoids	(Jiang et al., 2013)
	23	Naringenin	C_15_H_12_O_5_	0. 30	Flavonoids	(Ma et al., 2014)
	24	Isolariciresinol	C_20_H_24_O_6_	0. 60	Lignans	(Jiang et al., 2013)
	25	Methyl-3,4-dihydroxybenzoate	C_8_H_8_O_4_	0. 40	Phenolic acids	(Jiang et al., 2013)
	26	2-(2-Hydroxy-5-(methoxycarbonyl) phenoxy)benzoic acid	C_15_H_12_O_6_	1.68	Phenolic acids	(Jiang et al., 2013)
	27	Stigmasterol	C_29_H_48_O	0. 70	Sterols	(Jiang et al., 2013)
	28	Methylshikimate	C_8_H_12_O_5_	25.50	Esters	(Jiang et al., 2013)
	29	Ethyl shikimate	C_9_H_14_O_5_	3.75	Esters	(Jiang et al., 2013)
**Pulps**
	30	Propelargonidin	C_30_H_26_O_12_	/	Anthocyanins	(Lv et al., 2015)
	31	Prodelphinidin	C_45_H_36_O_21_	/	Anthocyanins	(Lv et al., 2015)
	32	Procyanidin	C_30_H_26_O_18_	/	Anthocyanins	(Lv et al., 2015)
	33	(+)-Catechin	C_15_H_14_O_6_	0.02–0.11	Flavonoids	(Zhang et al., 2013)
	19	(-)-Epicatechin (EC)	C_15_H_14_O_6_	2.31	Flavonoids	(Su et al., 2014)
	34	Quercetin-3-O-rutinoside-7-O-α-L-rhamnoside	C_33_H_40_O_20_	17.25	Flavonoids	(Su et al., 2014)
	35	(24R)-5α-stigmast-3, 6-dione	C29H48O2	/	Flavonoids	(Tu et al., 2002)
	36	Caffeic acid	C_9_H_8_O_4_	0.02–0.14	Phenolic acids	(Zhang et al., 2013)
	37	Chlorogenic acid	C_16_H_18_O_9_	0.06–0.18	Phenolic acids	(Zhang et al., 2013)
	38	5-Hydroxymethyl-2-furfurolaldehyde (5-HMF)	C_6_H_6_O_3_	0. 51	Furfurals	(Zhou et al., 2012)
	39	Benzyl alcohol	C_7_H_8_O	0. 15	Alcohols	(Zhou et al., 2012)
	40	Hydrobenzoin	C_14_H_14_O_2_	0. 99	Alcohols	(Zhou et al., 2012)
**Seeds**
	7	Procyanidin A2	C_30_H_24_O_12_	0.18	Anthocyanins	(Xu et al., 2010a)
	8	Proanthocyanidin A1	C_31_H_24_O_12_	0.14	Anthocyanins	(Xu et al., 2010a)
	41	Proanthocyanidin A6	C_31_H_28_O_12_	0.19	Anthocyanins	(Xu et al., 2010a)
	42	Aesculitannin A	C_45_H_36_O_18_	0.26	Anthocyanins	(Xu et al., 2010a)
	43	Litchitannin A1	C_45_H_34_O_18_	0.14	Anthocyanins	(Xu et al., 2010a)
	44	Litchitannin A2	C_45_H_34_O_18_	0.18	Anthocyanins	(Xu et al., 2010a)
	45	2α,3α-Epoxy-5,7,3`,4`-tetrahydroxyflavan-(4β-8-catechin)	C_30_H_24_O_12_	2.40	Anthocyanins	(Wang et al., 2011a)
	46	Epicatechin-(2β→O→7,4β→8)-epiafzelechin-(4α→8)-epicatechin	C_45_H_36_O_17_	0.29	Anthocyanins	(Xu et al., 2010b)
	47	2β,3β-Epoxy-5,7,3`,4`-tetrahydroxyflavan-(4α-8-epicatechin)	C_30_H_24_O_12_	1.07	Anthocyanins	(Wang et al., 2011a)
	48	2α,3α-Epoxy-5,7,3`,4`-tetrahydroxyflavan-(4β-8-epicatechin)	C_30_H_24_O_12_	3.52	Anthocyanins	(Wang et al., 2011a)
	49	Litchiol A	C_21_H_32_O_10_	0.37	Anthocyanins	(Wang et al., 2011a)
	50	Litchiol B	C_9_H_12_O_6_	0.07	Anthocyanins	(Wang et al., 2011a)
	51	(-)-Epicatechin-3-gallate (ECG)	C_22_H_18_O_0_	/	Anthocyanins	(Prasad et al., 2009)
	52	Epicatechin-(7,8-bc)-4β-(4hydroxyphenyl)-dihydro-2(3H)-pyranone	C_24_H_20_O_8_	0.29	Flavonoids	(Xu et al., 2010a)
	53	Quercetin	C_15_H_10_O_7_	/	Flavonoids	(Ren et al., 2013)
	54	Pinocembrin-7-O-[(2``,6``-di-O-α-L-rhamnopyranosyl)-β-D-glucopyranoside]	C_33_H_42_O_17_	/	Flavonoids	(Ren et al., 2013)
	55	(-)-Pinocembrin-7-O-neohesperidoside (Onychin)	C_27_H_32_O_13_	/	Flavonoids	(Ren et al., 2013)
	56	Kaempferol-7-O-neohesperidoside	C_27_H_30_O_15_	0.13	Flavonoids	(Xu et al., 2010a)
	57	Tamarixetin 3-O-rutinoside	C_28_H_32_O_16_	0.39	Flavonoids	(Xu et al., 2010a)
	58	Kaempferol-7-O-β-D-glucopyranoside	C_21_H_20_O_11_	/	Flavonoids	(Ren et al., 2013)
	59	Pinocembrin-7-O-glucoside	C_21_H_22_O_8_	/	Flavonoids	(Ren et al., 2013)
	60	(2S)-Pinocembrin-7-O-(6-O-α-L-rhamnopyranosyl-β-D-glucopyranoside)	C_27_H_32_O_13_	0. 03	Flavonoids	(Ren et al., 2011)
	61	Naringin	C_27_H_32_O_14_	0.30	Flavonoids	(Wang et al., 2011a)
	62	(2R)-Pinocembrin-7-neohesperidoside	C_27_H_32_O_13_	0.69	Flavonoids	(Wang et al., 2011a)
	63	Dihydrocharcone-4`-O-β-D-glucopyranoside	C_21_H_24_O_10_	0.57	Flavonoids	(Wang et al., 2011a)
	64	(2R)-Naringenin-7-O-(3-O-α-L-rhamnopyranosyl-β-D-glucopyranoside)	C_27_H_32_O_14_	0.08	Flavonoids	(Ren et al., 2011)
	65	Litchioside D	C_33_H_42_O_17_	0.28	Flavonoids	(Xu et al., 2010a)
	66	Pinocembrin-7-O-[(6``-O-β-D-glucopyranoside)-β-D-glucopyranoside]	C_27_H_32_O_14_	/	Flavonoids	(Ren et al., 2013)
	67	Narcissin	C_28_H_32_O_16_	/	Flavonoids	(Ren et al., 2013)
	68	Taxifolin-4`-O-β-D-glucopyranoside	C_21_H_22_O_13_	0.70	Flavonoids	(Xu et al., 2010a)
	69	(2S)-Pinocembrin-7-O-(6``-O-α-L-arabinosyl-β-D-glucopyranoside)	C_26_H_30_O_13_	/	Flavonoids	(Zhao et al., 2007)
	70	Phlorizin	C_21_H_24_O_10_	/	Flavonoids	(Ren et al., 2013)
	71	Scopoletin	C_10_H_8_O_4_	0.07	Coumarins	(Wang et al., 2011a)
	72	Protocatechuic acid (PA)	C_7_H_6_O_4_	0.43	Phenolic acids	(Wang et al., 2011a)
	73	Coumaric acid	C_9_H_8_O_3_	0.20	Phenolic acids	(Wang et al., 2011a)
	74	Gallic acid	C_7_H_6_O_5_	/	Phenolic acids	(Prasad et al., 2009)
	75	Butylated hydroxytoluene	C_14_H_22_O	3.80	Phenolic acids	(Jiang et al., 2013)
	76	Litchioside A	C_31_H_52_O_10_	0.23	Lignans	(Xu et al., 2011)
	77	Litchioside B	C_30_H_44_O_10_	0.10	Lignans	(Xu et al., 2010a)
	78	Pumilaside A	C_21_H_38_O_8_	0.39	Lignans	(Xu et al., 2010a)
	79	Funingensin A	C_21_H_36_O_7_	0.16	Lignans	(Xu et al., 2010a)
	80	Pterodontriol-D-6-O-β-D-glucopyranoside	C_21_H_38_O_18_	0.20	Lignans	(Wang et al., 2011a)
	81	Methyl dihydrosterculate	C_20_H_38_O_2_	/	Fatty Acids	(Stuart and Buist, 2004)
	82	2,5-Dihydroxy-hexanoic acid	C_6_H_12_O_4_	0.10	Fatty Acids	(Wang et al., 2011a)
	83	Litchioside C	C_19_H_34_O_9_	0.60	Fatty Acids	(Xu et al., 2011)
	84	3-Oxotrirucalla-7,24-dien-21-oic acid	C_30_H_46_O_3_	0.88	Triterpenes	(Tu et al., 2002)
	38	5-Hydroxymethyl-2-furfurolaldehyde (5-HMF)	C_6_H_6_O_3_	0. 51	Furfurals	(Zhou et al., 2012)
	39	Benzyl alcohol	C_7_H_8_O	0. 15	Alcohols	(Zhou et al., 2012)
	40	Hydrobenzoin	C_14_H_14_O_2_	0. 99	Alcohols	(Zhou et al., 2012)
**Leaves**
	7	Procyanidin A2	C_30_H_24_O_12_	2.00	Anthocyanins	(Sun et al., 2010)
	85	(-)-Pinocembrin 7-O-rutinoside	C_27_H_32_O_13_	0.15	Anthocyanins	(Xu et al., 2010a)
	86	Cinnamtannin B1	C_45_H_36_O_18_	1.18	Anthocyanins	(Wen et al., 2015)
	19	(-)-Epicatechin (EC)	C_15_H_14_O_6_	27.76	Flavonoids	(Wen et al., 2014a)
	87	Luteolin	C_15_H_10_O_6_	0.10	Flavonoids	(Wen et al., 2014a)
	88	Kaempferol-3-O-β-D-glucoside	C_21_H_20_O_11_	9.41	Flavonoids	(Wen et al., 2014a)
	89	Kaempferol-3-O-α-rhamnoside	C_21_H_20_O_10_	0.13	Flavonoids	(Wen et al., 2014a)
	17	Rutin	C_27_H_30_O_16_	0.67	Flavonoids	(Wen et al., 2014a)
	90	Litchtocotrienol A	C_27_H_42_O_4_	0. 16	Tocotrienols	(Lin et al., 2015)
	91	Litchtocotrienol B	C_28_H_44_O_5_	0. 16	Tocotrienols	(Lin et al., 2015)
	92	Litchtocotrienol C	C_28_H_44_O_4_	0.09	Tocotrienols	(Lin et al., 2015)
	93	Litchtocotrienol D	C_29_H_46_O_5_	0.04	Tocotrienols	(Lin et al., 2015)
	94	Litchtocotrienol E	C_27_H_40_O_3_	0.09	Tocotrienols	(Lin et al., 2015)
	95	Litchtocotrienol F	C_28_H_42_O_4_	0.04	Tocotrienols	(Lin et al., 2015)
	96	Litchtocotrienol G	C_28_H_42_O_5_	0.04	Tocotrienols	(Lin et al., 2015)
	97	Cyclolitchtocotrienol A	C_27_H_40_O_4_	0. 20	Tocotrienols	(Lin et al., 2015)
	98	Macrolitchtocotrienol A	C_27_H_39_O_3_	0.02	Tocotrienols	(Lin et al., 2015)
	99	Schizandriside	C_25_H_32_O_10_	0.94	Lignans	(Wen et al., 2014b)
	100	4,7,7`,8`,9,9`-Hexahydroxy-3,3`-dimethoxy-8,4`-oxyneolignan	C_20_H_26_O_9_	0.56	Lignans	(Wen et al., 2015)
	101	β-Sitosterol	C_29_H_50_O	/	Sterols	(Malik et al., 2010)
	102	Betulin	C_30_H_50_O_2_	/	Sterols	(Malik et al., 2010)
	103	Betulinic acid	C_29_H_48_O_3_	/	Sterols	(Malik et al., 2010)
	104	Lup-12,20(29)diene-3, 27-diol	C_30_H_48_O_2_	0.15	Triterpenes	(Malik et al., 2010)
	105	Lupeol	C_30_H_50_O	/	Triterpenes	(Malik et al., 2010)
	106	Secoisolariciresinol-9`-O-β-D-xyloside	C_25_H_34_O_10_	0.41	Glycosides	(Wen et al., 2015)
	107	Ehletianol C	C_30_H_36_O_10_	0.07	Glycosides	(Wen et al., 2014b)
	108	Sesquimarocanol	C_30_H_38_O_10_	0.61	Alcohols	(Wen et al., 2014b)
	109	Sesquipinsapol B	C_30_H_36_O_9_	0.10	Alcohols	(Wen et al., 2014b)
**Flowers**
	110	Gentisic acid	C_7_H_6_O_4_	/	Phenolic acids	(Ding et al., 2015)

### The Multi-Targeted Anticancer Effects of Litchi Ingredients

We summarized the confirmed anticancer ingredients of Litchi by going through each original published articles and found that 19 compounds (6 Anthocyanidins, 7 Flavonoids, 3 Phenolic acids, 2 Sterols, 1 Triterpenes) might inhibit cancer development through multifunctional mechanisms including regulation of cell proliferation, apoptosis, metabolism, metastasis, angiogenesis, stemness, and immunity. The anticancer ingredients with their corresponding effects, molecular targets, and cancer types were listed in **Table 2**. We then discovered that a single component could have a range of targets and different components had overlapping molecular targets, hence they formed a complicated regulatory network. In order to unravel this intricate web of interactions, we applied network pharmacology method to analyze the anticancer effects of Litchi from a perspective of system biology.

**Table 2:** The Anticancer Ingredients and Targets from Litchi.

Category	Ingredients	Effects	Targets	Cancer types	Ref
**Anthocyanins**	(-)-Epicatechin-3-gallate (ECG)	anti-proliferation	ATF3, CCNB1/D1/D3/E, CDC2/4/6, CDKN1A/1B/2B, GDF15, TP53, TRAF1	pancreatic and colon cancer	(Baek et al., 2004; Lim et al., 2006; Kürbitz et al., 2011; Cordero-Herrera et al., 2013)
		promoting apoptosis	AKT1, BAX, BCL2, CASP3, MAPK1, JNK, CDKN1A, TP53	colon cancer	(Cordero-Herrera et al., 2013)
		inhibiting metabolism	G6PD, TKT	colon cancer	(Saánchez-Tena et al., 2013)
	(-)-Epigallocatechin (EGC)	promoting apoptosis	BAX, BCL2	breast cancer	(Vergote et al., 2002)
	Epigallocatechin gallate (EGCG)	anti-proliferation	AKT1, AP1, APAF1, ATM, BIRC5, CCNB1, CDC2, CDKN1A, CYC, DIABLO, EGFR, ERBB2/3, FAS, FASLG, GDF15, JNK1/2, MAPK1/14/3, MLH1, MSH2, MTCO2, MYC, NFKB, PARP1, PEG2, PGE2, PIK3CA, PRKAA2, PTEN, PTK2, SP1, TP53, XRCC6	pancreatic, lung, colorectal, breast, prostate, gastric, and ovarian cancer	(Sukhthankar et al., 2010; Kuerbitz et al., 2011; Wang et al., 2011b; Lee et al., 2012; Luo et al., 2014; Fu et al., 2019)
		attenuating angiogenesis	MPI, ACTA1, PECAM1	lung cancer	(Deng et al., 2019)
		inhibiting metastasis	CDH1/2, CSRC, ERBB2, ESR2, MAPK8, MIR138, NFKB, PTK2, SLC22A5, SNAIL1/2, TJP1, VIM,	lung, colon, breast, bladder, and prostate cancer	(Shimizu et al., 2005a; Punathil et al., 2008; Sen et al., 2010; Sen and Chatterjee, 2011; Deng and Lin, 2011; Ko et al., 2013; Mukherjee et al., 2014; Li et al., 2015; Huang et al., 2016b; Luo et al., 2017)
		promoting apoptosis and autophagy	AKT1, AP1, APAF1, ATM, BAD, BAX, BCL2/L1, BIRC5, CASP2/3/8/9, CCNB1, CDC2, CDKN1A, CYC, DIABLO, EGFR, ERBB2/3, FAS, FASLG, GDF15, JNK1/2, KRAS, MAPK1/14, MLH1, MSH2, MTCO2, MYC, NFKB, PARP1, PEG2, PGE2, PIK3CA, PRKAA2, PTEN, PTK2, SP1, TP53	pancreatic, lung, colon, head and neck, breast, and bladder cancer	(Shimizu et al., 2005b; Park et al., 2009; Deng and Lin, 2011; Kang et al., 2013; Cerezo-Guisado et al., 2015; Li et al., 2016; Luo et al., 2017; Huang et al., 2017; Ni et al., 2018; Gu et al., 2018; Wei et al., 2018; Lu et al., 2019)
		sensitizing chemotherapy	ABCG2/B1, ATP7A/B, BAX, BBC3, CASP3/7/9, CDKN1A, ERCC1, GSTP1, HDGF, HSPA5, IRS1, JAK3, LC3II, MAPK1, MIR155, NFKB, PARP1, PDZD3/K1, PGP, PMAIP1, RPS6KB1, RXRA, SFN, SLC22A5, SLC31A1/A2, STAT3, TFAP2A, VEGFA	colorectal, oral, gastric, pancreatic, lung, ovarian, and cervical cancer	(Tang et al., 2012; Hu et al., 2015; Wang et al., 2015; Flores-Pérez et al., 2016; Tang et al., 2017; Heyza et al., 2018; McDonnell et al., 2019; Pons-Fuster Lopez et al., 2019)
		inhibiting metabolism	HIF1A, HK2, LDHA, PFKM, PKM, SLC2A1, VEGFA	breast cancer	(Zhu et al., 2017; Wei et al., 2018)
		suppressing stemness	ALDH1A1, CD44, CTNNB1, GSK3B, MYC, NANOG, OCT4, PROM1, STAT3	nasopharyngeal and lung cancer	(Lin et al., 2014)
	Proanthocyanidin B2	promoting apoptosis	AKT1, ATG5, BAX, BECN1, CASP3, LC3I/II, MTOR, PIK3CA	colorectal cancer	(Zhang et al., 2019b)
	Cyanidin-3-glucoside	anti-proliferation	MKI67, MUCIN4	ovarian cancer	(Zeng et al., 2012)
		attenuating angiogenesis	STAT3, VEGFA	breast cancer	(Ma and Ning, 2019)
		inhibiting metastasis	CDH1/2, CJUN, CSRC, ERBB2, ESR2, MAPK8, MIR138, MMP2, NFKB, PLAU, PTK2, SLC22A5, SNAIL1/2, TIMP2, TJP1, VIM,	breast and lung cancer	(Xu et al., 2010c; Liang et al., 2019)
		promoting apoptosis	BCL2, CASP3	breast cancer	(Cho et al., 2017)
	Cyanidin-3-rutinoside	inhibiting metastasis	MMP2, PLAU, TIMP2, CJUN, NFKB,	lung cancer	(Chen et al., 2006)
**Flavonoids**	(+)-Catechin	anti-proliferation	CCNA/B1, CDK2/4, JNK, MAPK1/9/14	breast cancer	(Deguchi et al., 2002)
		promoting apoptosis	CASP3/8/9, TP53	cervical cancer	(Al-Hazzani and Alshatwi, 2011)
		sensitizing chemotherapy	SOD2, GSTA1	pancreatic cancer	(Michel et al., 2018)
	(-)-Epicatechin (EC)	anti-proliferation	AKT1, MAPK1, NFKB, RAS	lung and pancreatic cancer	(Siddique et al., 2012; Varela-Castillo et al., 2018)
		promoting apoptosis	BAX, BCL2, NFKB, CDKN1A	colon cancer and lymphoma	(Mackenzie and Oteiza, 2006; Kim et al., 2012a)
		sensitizing radiotherapy and chemotherapy	CHEK2, CDKN1A, CASP3, GADD45A, DDIT3	pancreatic, lung cancer, and glioblastoma	(Saha et al., 2010; Elbaz et al., 2014)
	Kaempferol	anti-proliferation	AKT1, CCNA/B1/D1/E, CDC2/25C, CDK1/2/20/4/5R1, CDKN1A, CHEK1/2, CMET, DNMT3B, ERBB3, ERRA, ERRG, GTF2H2, HIF1A, IGF1/1R, MAP1LC3A, MAPK1/14/3, MCL1, MIR21/340, MTOR, PIK3CA/R1, PRKAA2, PTEN, SQSTM1, TP53, USF2	bladder, breast, cervical, lung, colon, gastric, and liver cancer	(Choi and Ahn, 2008; Li et al., 2009; Mylonis et al., 2010; Wang et al., 2013; Cho and Park, 2013; Huang et al., 2013; Lee et al., 2014a; Dang et al., 2015; Kim et al., 2016; Qiu et al., 2017; Drouet et al., 2018; Han et al., 2018a; Wu et al., 2018; Zhu et al., 2018; Zhang and Ma, 2019)
		attenuating angiogenesis	AKT1, ESRRA, HIF1A, VEGFA	ovarian cancer	(Luo et al., 2009)
		inhibiting metastasis	AKT1, CDH1/2, CJUN, MAPK2/3, MIR21, MMP2/9, MTOR, MYC, PIK3CA, PTEN, PTK2, RAC1, RHOA, SMAD3, SNAIL1, VIM	breast, oral cancer, lung, liver, and renal carcinoma	(Lin et al., 2013; Jo et al., 2015) (Lee et al., 2017a; Hung et al., 2017; Zhu et al., 2018)
		promoting apoptosis and autophagy	AKT1, ATG7, ATM, BAD, BAX, BCL2/L1, BECN1, BID, BIK, CASP3/7/8/9, CFLIP, CYC, DDIT3, EHMT2, EREG, ERN1, FAS, H2AX, JNK, LC3I/II/III, MAP2K1/2, MAPK1/3, MTCO2, TERT, TNFRSF10A	bladder, breast, cervical, colon, colorectal, endometrial, gastric, lung, and ovarian cancer	(Nguyen et al., 2003; Li et al., 2009; Luo et al., 2011; Xie et al., 2013; Lee et al., 2014b; Kim et al., 2016; Yi et al., 2016; Kashafi et al., 2017; Zhao et al., 2017; Choi et al., 2018; Chuwa et al., 2018; Kim et al., 2018; Zhu et al., 2018; Zhang and Ma, 2019)
		sensitizing chemotherapy	ABCC6, AKT1, BAX, BCL2/L1, BIRC5, CASP3/7/8/9/10, CDKN1A, FAS, JAK1, JNK, MAPK1/14, MYC, NFKB, PARP1, PIK3CA, ROS1, STAT3, TNFRSF10A, XIAP,	ovarian, lung, and colorectal cancer	(Luo et al., 2010; Kuo et al., 2015; Riahi-Chebbi et al., 2019)
		inhibiting metabolism	SLC2A1/16A1	breast cancer	(Azevedo et al., 2015)
		enhancing immunity	CSF2, MAP2K1, MAPK2/3, PKC, PLC	prostate cancer	(Bandyopadhyay et al., 2008)
	Luteolin	attenuating angiogenesis	NOTCH1, VEGFA	gastric cancer	(Zang et al., 2017a)
		inhibiting metastasis	AKT1, CDH1/2, CTNNB1, CYCD1, HES1, HEY1, MIR384, MMP2/3/7/9/16, NFKB, NFKBIA, NOTCH1, PIK3CA, PTN, SNAIL1/2, STAT3, VIM	gastric, pancreatic, breast, lung, and colorectal cancer	(Chen et al., 2013; Huang et al., 2015b; Lin et al., 2017; Zang et al., 2017b; Yao et al., 2019)
		promoting apoptosis	AIF, AKT1, ANO1, AURKB, BANF1, BAX, BCL2/L1, BIRC5, CASP3/9, CCND1/E, CDKN1A, DEDD2, ENG, GAK, HSP90, HTERT, MAPK1/14/3, MCL1, MCL4, MIR107/139/155/21/224/301/340/34A/422A/5703/630, MTOR, MYC, NFE2L2, NFKBIA, NOTCH1, PIK3CA, PTPN6, SIRT1, STAT3, TET1, TP53, VRK1,	breast, colon, gastric, lung, pancreatic, and prostate cancer	(Kim et al., 2012b; Ma et al., 2015; Han et al., 2016; Song et al., 2017; Seo et al., 2017; Li et al., 2018; Jiang et al., 2018; Kang et al., 2019)
		sensitizing chemotherapy	BAX, BCL2/L1, CASP3/7/8/9, CCNE2, CDH2, FAS, GSTA1/2, HMOX1, JAK1, JNK, MAPK1/14, NFE2L2, NFKBIA, PARP1, PPARG, PRKAR2A, PTK2, PTPN11, RACK1, RELA, ROS1, SLC22A5, SNAIL1/2, STAT1/2/3, TWIST1, TYK2, VIM	ovarian, lung, colorectal, cervical, breast, ovarian, and liver cancer	(Tu et al., 2013; Chian et al., 2014; Qu et al., 2014; Tai et al., 2014; Yang et al., 2014; Cho et al., 2015; Dia and Pangloli, 2017; Wang et al., 2018a; Liu et al., 2018)
		suppressing stemness	BMI1, CCND1, CD44, FZD6, IL6, MYC, OCT4, PROM1, STAT3	prostate and oral cancer	(Tu et al., 2016; Han et al., 2018b)
	Naringenin	anti-proliferation	CCND1, EREG, ESR2, MAPK1/14	cervical, colon, colorectal cancer, and hepatocarcinoma	(Totta et al., 2004; Song et al., 2015; Zhang et al., 2016)
		inhibiting metastasis	AKT1, CDH1, MAPK1/4, MMP2/9, NCL, NFKB, PKCZ, PKCE, RAC1, RHO, RHOA, SCN9A, SNAIL1/2, TGFB1, TWIST1, VIM	breast, lung, and bladder cancer	(Liao et al., 2014; Zhang et al., 2016; Chang et al., 2017; Aktas and Akgun, 2018; Han et al., 2018c; Zhao et al., 2019b)
		promoting apoptosis	AKT1, MAP3K5, ATF3, BAX, BCL2, BIRC5, CASP3/9, JNK, MAPK1/3/14, TP53, RPS6KB1, ROS1, RPS6	prostate, pancreatic, colon, breast, and gastric cancer	(Song et al., 2016; Lim et al., 2017; Park et al., 2017; Wang et al., 2019a)
		sensitizing chemotherapy	CDKN2A, BCL2, CASP3/9, BAX, PTK2, MAPK14	lung and pancreatic cancer	(Parashar et al., 2018; Lee et al., 2019)
		inhibiting metabolism	AKT1, GTF2H2, MAP2K1/2, MAPK1, NFKB1, PIK3CA	breast cancer	(Harmon and Patel, 2004)
		enhancing immunity	GZMB, ID2, IFNG, IRF2, SMAD3/7	lung cancer and melanoma	(Lian et al., 2018)
	Naringin	anti-proliferation	AKT1, BIRC5, CDKN1A, CTNNB1, EGFR, MAPK1, MIR126, MTOR, NFKB, PIK3CA, VCAM1	lung, cervical, gastric, and breast cancer	(Li et al., 2013; Raha et al., 2015; Yoshinaga et al., 2016; Chen et al., 2018a)
		promoting apoptosis	BAX, CASP1/3/9, FADD, FAS, MTCO2, NFKB, TP53, CDKL2	cervical and lung cancer	(Ramesh and Alshatwi, 2013; Zeng et al., 2014)
		sensitizing chemotherapy	ChIL3, BAX, BID, BIRC5/7, CASP3, CDKN1A/B, CYC, MRC1NFKB, PGP, TP53	breast, prostate, and ovarian cancer	(Zhang et al., 2015; Aktas and Akgun, 2018; Erdogan et al., 2018)
	Rutin	sensitizing chemotherapy	PGP, ABCG2,	breast cancer	(Iriti et al., 2017)
		promoting apoptosis	BAX, BCL2, CASP3/8/9, GSK3B, IKKB, CHUK, MK2, NFKB, MAPK14, TP53, PARP1, TNF	colon and lung cancer	(Guon and Chung, 2016; Wu et al., 2017; Nafees et al., 2018)
		suppressing stemness	NANOG, POU5F1, SOX2	lung cancer	(Yamagata et al., 2018)
**Phenolic acids**	Chlorogenic acid	anti-proliferation	MAPK1, ROS1	colon cancer	(Hou et al., 2017)
		promoting apoptosis	AKT1, CCNA/B1/D1/D3/E, CDC2/25C, CDK1/2/4/6, CHEK1/2, CDKN2A/2B/1A/1B, MAPK1/8/14, PIK3CA, SKP2, BAX, BCL2, CASP3	bladder, prostate, renal carcinoma, and breast cancer	(Deka et al., 2017; Yamagata et al., 2018; Wang et al., 2019b)
	Gallic acid	anti-proliferation	AKT1, CCNA/B1/D1/D3/E, CDC25C/2C, CDK2/4/6, CDKN1A/1B, CHEK1/2, INK4, MAPK1/14, P18, PIK3CA, SKP2	bladder, prostate, and breast cancer	(Hsu et al., 2011; Lee et al., 2017b; Liao et al., 2018; Sales et al., 2018)
		attenuating angiogenesis	AKT1, EGFR, HIF1A, MAPK1, PTEN, VEGFA	cervical and ovarian cancer	(He et al., 2016; Sales et al., 2018)
		inhibiting metastasis	AKT1, CDC42, CHUK, CJUN, EGFR, GRB2, IL6, MAPK81/2, JUN, MAPK2/3/14, MEKK3, MMP2/9, NFKB, PIK3CA, PKC, PTK2, RAC1, RAS, RELA, RHOA, RHOB, ROS1, SOS1, SRC, STAT3	oral, prostate, bladder, breast, and gastric cancer	(Ho et al., 2010; Ho et al., 2013; Kuo et al., 2014; Heidarian et al., 2016; Chen et al., 2016)
		promoting apoptosis	AKT1, APAF1, ATM, ATR, BAK1, BAX, BCL2/L1, BIK, BRCA1, CASP3/8/9, CKII, CYC, EREG, GSH, H2AX, JNK, MDC1, MGMT, MTOR, PARP1, PRKDC, ROS1, RPS6KB1, TP53, XIAP	oral, prostate, pancreatic, cervical, lung, and esophageal cancer	(Faried et al., 2007; Chen et al., 2009; You et al., 2010; Russell et al., 2012; Liu et al., 2012a; Lu et al., 2016; Lin and Chen, 2017)
		sensitizing chemotherapy	APAF1, BAX, BCL2, CASP3, CCNA/B, CCND1, DIABLO, EGFR, HIF1A, IL6, JAK1, MTCO2, MYC, NOS2, PARP1, ROS1, SRC, STAT3, TP53, VEGFA, XIAP	lung and cervical cancer	(Phan et al., 2016; Wang et al., 2016a; Aborehab and Osama, 2019)
	Protocatechuic acid (PA)	anti-proliferation	FGF2, JNK, MAPK3/1/14, NFKB1, PTK2, RELA,	lung cancer	(Tsao et al., 2014)
		inhibiting metastasis	AKT1, CDC42, CJUN, CXCL8, FGF2, FN1, IL6, MMP2/9, NCL, NFKB/IA, PIK3CA, PKCE, RAC1, RAS, RHOA/B, USF2, VEGFA	breast, lung, liver, cervical, and prostate cancer	(Yin et al., 2009; Lv et al., 2019)
		promoting apoptosis and autophagy	BAX, BCL2, CASP3, LC3I/II, PARP1	lung and ovarian cancer	(Tsao et al., 2014; Xie et al., 2018)
**Sterols**	Betulinic acid	anti-proliferation	MIR27A, SP1, YY1, ZBTB10	lung and breast cancer	(Hsu et al., 2012b; Liu et al., 2012b)
		attenuating angiogenesis	HIF1A, STAT3	prostate cancer	(Lu et al., 2018)
		promoting apoptosis	AKT1, BAD, BAK1, BAX, BCL2, CDH1, CASP3/9, CYC, NFKBIB, CHUK, MKI67, PMAIP1, CDKN1A/1B, TP53, CDKL2, PARP1, PIK3CA, ROS1, TIMP2, XIAP	colon, gastric, colorectal, cervical, prostate, and pancreatic cancer	(Shankar et al., 2017; Zeng et al., 2019)
		sensitizing chemotherapy	BAX, BCL2, BIRC5, CASP12/3, CDK6, CTNNB1, DDIT3, EGFR, EIF2A, GSK3B, HK2, HSPA5, MAP1LC3B, MAPK1, PARP1, RB1, SQSTM1, STAT3, TYMS, VDAC1	breast and lung cancer	(Ko et al., 2018; Cai et al., 2018; Wang et al., 2019c)
		inhibiting metabolism	CAV1, IKBA, LDHA/B, MYC, PDK1, RELA	breast cancer	(Jiao et al., 2019; Zeng et al., 2019)
		suppressing stemness	NANOG, OCT4, PRKAA2, SOX2	pancreatic cancer	(Sun et al., 2019)
	β-Sitosterol	promoting apoptosis	BAX, BCL2, CASP3	gastric cancer	(Zhao et al., 2009)
		sensitizing chemotherapy	AKT1, GSK3B, RELA, BAX, BCL2, SNAIL1, VIM	pancreatic cancer	(Cao et al., 2018)
**Triterpenes**	Lupeol	anti-proliferation	AKT1, BCL2, CCNA2/B/D3, CDC2/26C, CDK2/N1A/N1B/N2A, CLAUDIN1, CTNNB1, MAPK1, MYC, PLK1, TCF4, TP53	head and neck, colorectal, prostate, pancreatic, and cervical cancer	(Liu et al., 2015; Bhattacharyya et al., 2017; Emanuele et al., 2018; Wang et al., 2018b)
		inhibiting metastasis	BCL2, CLAUDIN1, MMP2/9, MTCO2, NFKB, RELA, TP53	colorectal and breast cancer	(Wang et al., 2016b; Wang et al., 2018c)
		promoting apoptosis	APAF1, BAX, BCL2, CASP3/9, EGFR, MKI67, PARP1, STAT3	cervical, head and neck, lung, and prostate cancer	(Prasad et al., 2008; Bhattacharyya et al., 2017; Min et al., 2019)
		sensitizing chemotherapy	ABCG2, MAPK1, EIF2A, CASP3	colon cancer	(Chen et al., 2018b)
		enhancing immunity	AKT1, BCL2, CTNNB1, IFNG, LAMP1, MAPK2/3, PIK3CA, PRF1	gastric cancer	(Wu et al., 2013)

#### Inhibition of Cancer Cell Proliferation

Sustained proliferation is a hallmark of cancer cells, and the restoration of dysregulated signaling pathways has always been a target for cancer treatment. The extracts from Litchi peels, pulps, seeds, leaves have been shown to inhibit the proliferation of a variety of cancer cells (Huang et al., 2015a; Gong et al., 2018; Zhao et al., 2019a). The 13 anti-proliferative compounds identified from Litchi and 100 regulated targets were summarized in **Table S1**. The detailed analysis of the top active ingredients, corresponding targets, and signal pathways affected was shown in **Figure 1**.

In total, this ingredient-target network (**Figure 1A**) was consisted of 113 nodes (**Table S1**) and the mean degree of all nodes in the network was 3.080. Overall, 3 out of the 13 anticancer compounds (**Figure 1A**) had high degree distributions (kaempferol: degree=39, Epigallocatechin gallate (EGCG): degree=36, gallic acid: degree=22) and all of them modulated more than 20 targets, which marked their pharmacological importance. Notably, those targets have more than one regulator (**Table S2**). Apart from 1 target that was regulated by 10 ingredients, 4 targets were regulated by over 5 ingredients and 28 targets were regulated by 2–4 ingredients (**Figure 1B**). Further, the 4 top targets (MAPK1, CDKN1A, MAPK14, AKT1) were screened out from **Figures 1A, B**, whose degree values were more than two folds of the median degree of all nodes in the network. This suggested that multiple ingredients could potentially exert synergistic anti-proliferation effects. In particular, the interactions among the above 4 top targets and Litchi ingredients (**Table S3**) were analyzed in **Figure 1C**. With the results shown in **Figure 1C**, we could conclude that there were 11 out of 13 ingredients that could regulate the top targets with anti-proliferative effects. It was also confirmed that the top 4 targets played an important role in the anti-proliferative process. Particularly, kaempferol, EGCG, and gallic acid could regulate all the top targets, and this conclusion was similar to that in **Figure 1A** where 3 ingredients mentioned above had outstanding pharmacological significance. To further clarify the anticancer mechanism of Litchi ingredients, the pathway enrichment analysis based on above 4 top targets was performed. There were 63 signaling pathways involved in the anti-proliferation effects of Litchi ingredients (**Figure 1C** and **Table S3**), and FoxO, VEGF, Prolactin, ErbB, HIF-1, Toll-like receptor, TNF, Rap1, MAPK, and PI3K-Akt signaling pathways were the top 10 pathways according to their P values (**Figure 1D**). All of the 4 top targets were elements of FoxO signaling pathway and 3 out of the top 4 targets were elements of other 9 top pathways. It indicated that these top 10 pathways might be the major signaling pathways that are responsible for the anti-proliferation effects of Litchi.

#### Induction of Cancer Cell Apoptosis and Autophagy

Apart from uncontrollable proliferation, resistance to cell death is another strategy employed by cancer cells to fuel its growth. Cancer cells have evolved a series of strategies to inhibit cell death while Litchi ingredients have been reported to have pro-apoptosis and pro-autophagy effects (Hsu et al., 2012a; Emanuele et al., 2018). Hence, we summarized data from literature and constructed the network (**Figure 2A**) based on 18 ingredients from Litchi and 138 targets (**Table S4**) which related to cell apoptosis and autophagy. The network was consisted of 156 nodes and 283 edges altogether, representing the extensive interactions among 18 ingredients and 138 targets (**Table S4**). Not surprisingly, we found that the mean degree of node was 3.679 based on the topological analysis, suggesting that it was common for ingredients to have multiple targets. By referring to the mean degree, we identified 6 top ingredients with a median degree ≥20, namely luteolin, EGCG, kaempferol, gallic acid, betulinic acid, and chlorogenic acid, with the top 2 having over 40 targets. Hence, we concluded that those top 6 ingredients were likely to be crucial components in promoting apoptosis and autophagy. Further, in order to clearly elucidate if these targets were regulated by multiple ingredients, another analysis was performed in **Figure 2B**, which showed that there were 3 targets regulated by over 10 ingredients, 9 targets were regulated by 5–10 ingredients and 33 targets were regulated by more than 2 ingredients (**Figure 2B** and **Table S2**). From **Figures 2A, B**, we next screened out the top 6 targets (BAX, BCL2, CASP3, CASP9, TP53, AKT1) based on their degrees in the ingredient-target network. As shown in **Figure 2C** and **Table S5**, all of the top 6 targets could be regulated by luteolin and EGCG, and this implied that they had multiple anticancer activities. In addition, all the 18 ingredients involving in apoptosis and autophagy interacted with the top targets, which consolidated the importance of these top targets. KEGG enrichment analysis based on these 6 top targets showed that 39 signaling pathways were involved in the effects of inducing cancer cell apoptosis and autophagy (**Figure 2C** and **Table S5**), while p53, Neurotrophin, Sphingolipid, PI3K-Akt, Thyroid hormone, MAPK, VEGF, HIF-1, TNF signaling pathway and Adrenergic signaling in cardiomyocytes were the top 10 pathways (**Figure 2D**). Four out of these top 6 targets were elements of p53, Neurotrophin, Sphingolipid, and PI3K-Akt signaling pathways, which indicates that these four signaling pathways might be the major pathways responsible for anticancer effect by inducing apoptosis and autophagy.

#### Inhibiting Metastasis

Metastasis is another target in cancer therapeutic development due to its lethality (Liu et al., 2017). Litchi seed extracts could attenuate migration and invasion capabilities of PC3 and DU145 cells (Guo et al., 2017). Nine anti-metastasis ingredients of Litchi and 99 corresponding targets were listed in **Table S6**, the interaction network of which was shown in **Figure 3A**. We found that the mean degree of nodes in the network was 3.296. Then we screened out 4 top ingredients, namely EGCG, gallic acid, luteolin, and PA, with a median ≥20 degrees, which acted on 41, 29, 22, and 21 targets respectively. Therefore these 4 top ingredients identified were likely to be crucial bioactive components to inhibit metastasis. In addition, among the 99 targets, the network showed that MMP2 had the largest number of ingredient-target interactions (degree value of 8), followed by MMP9 (degree value of 7), making them likely to perform anti-metastasis functions. The remaining targets with lower degree and less than two folds of the mean degree of all nodes were also included. Then, the targets regulated by multiple ingredients were analyzed with a similar approach for more information. As shown in **Figure 3B** and **Table S2**, MMP2 and MMP9 were regulated by 8 and 7 ingredients respectively, followed by another 6 targets regulated by up to 5 ingredients and 26 targets regulated by 2 to 4 ingredients. The “ingredients-top targets-pathways” network (**Figure 3C** and **Table S7**) was constructed for the purpose of confirming the significance of top 2 targets, and this network indicated that as much as 8 ingredients exerted the anti-metastasis function through modulating MMP2 and MMP9. However, the signaling pathways enriched by KEGG based on 2 top targets merely included bladder cancer, estrogen signaling pathway, leukocyte transendothelial migration, proteoglycans in cancer and pathways in cancer. Both the top 2 targets were elements of these 5 pathways (**Figures 3C, D** and **Table S6**), which indicated these 5 pathways might be the key anti-metastasis mechanism of Litchi.

#### Sensitizing Chemotherapy and Radiotherapy

Chemotherapy and radiotherapy are two of the most common cancer treatments. Despite their clinical efficacy in clearing cancer cells, therapeutic resistance often inevitably occurs. Another reported effect of Litchi was that it sensitized chemotherapy and radiotherapy. Here we identified 12 compounds from Litchi and 106 corresponding molecular targets responsible for this function (**Table S8**), with the detailed interactions of the top ingredients, targets and signal pathways shown in the **Figure 4**. From **Figure 4A**, we screened out 5 top ingredients with a median degree ≥20, including luteolin, EGCG, kaempferol, gallic acid, and betulinic acid, which linked to as much as 35, 34, 25, 22, and 21 targets respectively. Not surprisingly, the mode of “multi-ingredients, multi-targets” was confirmed again by identifying CASP3, BAX, and BCL2 as the top targets, which had the degree values of 9, 8, 6 respectively, which were more than two folds of the median degree of all nodes in the network. In addition, there were another 32 targets regulated by more than 2 ingredients (**Figure 4B** and **Table S2**), which implied that Litchi ingredients could overcome chemo- and radio-resistance through a “multi-compounds, multi-targets” mode with potential synergistic effects. The “ingredients-top targets-pathways” network (**Table S9**) confirmed the importance of CASP3, BAX, and BCL2 further. In **Figure 4C**, 10 out of 12 ingredients that were involved in sensitizing chemotherapy and radiotherapy exerted anticancer activity through regulating the 3 top targets. Moreover, KEGG enrichment analysis of top 3 targets showed that 15 signaling pathways were involved in the chemotherapy and radiotherapy sensitization (**Figure 4C** and **Table S9**). All of the top 3 targets were elements of Amyotrophic lateral sclerosis (ALS), Colorectal cancer, Apoptosis, Hepatitis B, Tuberculosis and pathways in cancer, and 2 out of the top 3 targets were elements of p53 signaling pathway, Toxoplasmosis, Sphingolipid, and Neurotrophin signaling pathway, which indicates that the 10 pathways mentioned above might be responsible for the anticancer effect of Litchi on chemotherapy and radiotherapy sensitization (**Figure 4D**).

#### Other Anticancer Effects

Apart from the four effects exerted by Litchi ingredients for the major anticancer functions as listed above, several other targets were also found to be involved in the suppression of cancer stemness, metabolism, and angiogenesis, while also in the enhancement of immunity as listed in **Table S10**. However, the experiments validations on the anticancer effect of Litchi ingredients from these four aspects were very limited. Therefore, we only constructed a simple ingredient-target network map (**Figure 5**). The results showed that these mechanisms involved a total of 10 active ingredients, among which 5 belonged to the top ingredients from the previous screening including betulinic acid, EGCG, luteolin, gallic acid, and kaempferol, which further illustrated their importance. At the same time, we suggest that the remaining 5 ingredients (chlorogenic acid, (-)-Epicatechin-3-gallate (ECG), naringenin, cyanidin-3-glucoside, lupeol) and their detailed mechanisms need to be further explored.

## Discussion

Numerous studies have shown that Litchi contains a variety of anti-cancer ingredients, which act by multiple targeting. Emanuele and Ibrahim described Litchi’s nutritional value and reviewed the anti-tumor components and targets of Litchi with detailed listing but lacked a systematic analysis (Ibrahim and Mohamed, 2015; Emanuele et al., 2017). In the present study, we collected 110 compounds isolated from Litchi and found 19 components with anticancer effects based on 241 published research papers. The detailed information for each one of these compounds was listed in **Tables 1** and **2** with corresponding targets. Then the network pharmacology approach was applied to explore the complicated “multi-ingredients, multi-targets, multi-pathways” anticancer mechanisms of Litchi from a system biology perspective.

**Figure 6:**
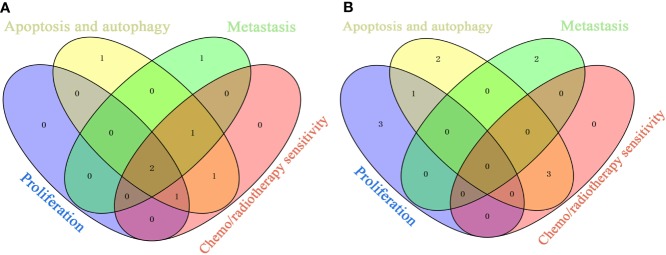
Overlaps of Top Ingredients and Targets Related to Anti-Proliferation, Inducing Apoptosis and Autophagy, Inhibiting Metastasis and Sensitizing. **(A)** Top ingredients. **(B)** Top targets.

**Figure 7:**
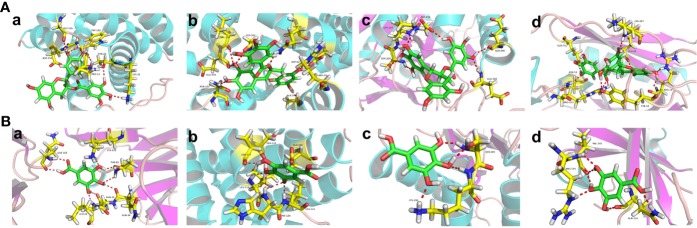
The Binding Modes of Top Ingredients and Top Targets. **(A)** The binding modes of EGCG in the active pockets: a (BAX/PDB ID: 6EB6); b (BCL2/PDB ID: 4AQ3); c (CASP3/PDB ID: 6CL0); d (AKT1/PDB ID: 6HHG). **(B)** The binding modes of gallic acid in the active pockets: a (BAX/PDB ID: 5W5Z); b (BCL2/PDB ID: 5VAX); c (CASP3/PDB ID: 6CL0); d (AKT1/PDB ID: 6BUU).

We identified the top ingredients, top targets, and top signaling pathways of Litchi with anticancer effect from four major aspects including anti-proliferation, cell death promotion, inhibition of metastasis, and sensitization of chemotherapy and radiotherapy. Further, in order to identify the primary ingredients and targets acting on all four anticancer functions listed above, we performed analysis (**Figure 6** and **Table S11**) and found EGCG and gallic acid to be the top ingredients participating in all of the four anticancer functions (**Figure 6A** and **Table S11**). Moreover, EGCG was also involved in the suppression of cancer stemness, cancer metabolism, and angiogenesis, while gallic acid was involved in attenuating angiogenesis (**Table S10**). These results suggest that they are likely to be the major anticancer ingredients in Litchi. Apart from that, we also found that kaempferol, luteolin, and betulinic acid were the top ingredients which carried out at least 2 of anticancer mechanisms (**Figure 6A** and **Table S11**). After selecting the primary ingredients from the overlapping parts, we found that BAX, BCL2, and CASP3 were the common targets which could induce apoptosis, autophagy, and sensitization, while AKT1 was a common target to suppress proliferation and induce apoptosis (**Figure 6B** and **Table S11**). To further study the interactions among top ingredients (EGCG and gallic acid) and top targets (BAX, BCL2, CASP3, and AKT1), a molecular docking study was carried out to elucidate their binding modes. The result indicated a high binding affinity between EGCG and 4 targets with all of their total score greater than 6. However, gallic acid showed a lower binding affinity with each of their total score less than 6, while, only 2 top targets had active binding pockets for gallic acid with a total score of more than 5 (**Figure 7** and **Table S12**). We speculated that gallic acid might exert anticancer effects by indirectly interacting with the top targets. Other than identifying single ingredient and its corresponding effect or vice versa, we mapped the complex interactive network of the primary targets and ingredients from Litchi (**Table S11**). The results could be used to maximize the effects of Litchi ingredients by extracting only the identified functional components based on the principles of Component Formula, which is a new model to develop innovative TCM with the understanding of the effective ingredients and pharmacological mechanisms (Zhang and Wang, 2005). Notably, we have also found that some of the top pathways screened out in this study have been experimentally verified, such as PI3K-Akt, Ras and MAPK signaling pathways etc. (Lin et al., 2011; Wang et al., 2011a; Lim et al., 2017). Hence, we have collected and summarized the results from independent studies, and also investigated further into the complex network of the multiple active ingredients and targets of Litchi. This would help to guide people to further explore the potential cancer therapy values of Litchi.

This study systematically explored the anti-cancer mechanisms of Litchi using network pharmacology methods. However, it was distinct from traditional network pharmacology research, in which, the components and targets of a natural herb were mainly predicted based on online databases, followed by experimental verification *in vitro* and *in vivo*. In contrast, in this study, experiments were not of necessity because the anti-cancer ingredients, targets, and their interactions have already been experimentally confirmed in published literature. Furthermore, we collected information from independent studies and transformed them into a systematic interaction network with further analysis of the top ingredients, top targets and possible signaling pathways. For the first time, the anti-cancer properties of Litchi were explored from a new “multi-ingredients, multi-targets, and multi-pathways” perspective. However, selecting the top ingredients and top targets by network pharmacological methods alone has limitations, such as that it could neither reflect the anticancer effect intensity of these top ingredients, nor indicate if there was a correlation between the effectiveness of the ingredients and their concentrations. Also, we could not compare the pharmacokinetic parameters which directly affect drug efficacy. Therefore, based on the results of this article, we would use these top ingredients as a “Component Formula” in a combinatory manner and to explore their anti-cancer effect with *in vitro* and *in vivo* experiments in the follow-up studies.

## Data Availability Statement

All datasets generated for this study are included in the article/**Supplementary Material**.

## Author Contributions

HG, SC, and ZS designed this work. SC, YH, and YC drafted the manuscript. HG, YH, and DZ performed the network pharmacology analysis. QL made the figures. All authors read and approved the final version.

## Funding

This work was supported by the National Natural Science Foundation of China (81660681), Natural Science Foundation of Guangxi Province of China (2018GXNSFAA294080, 2020GXNSFAA259030), Guangxi First-class Discipline Project for Pharmaceutical Sciences (GXFCDP-PS-2018), Guangxi and Nanning Science and Technology Development Project of China (1598013-6, 20163151, 20155176).

## Conflict of Interest

The authors declare that the research was conducted in the absence of any commercial or financial relationships that could be construed as a potential conflict of interest.

## Abbreviations

See **Table S13**.
